# Mechanical Properties of a Newly Additive Manufactured Implant Material Based on Ti-42Nb

**DOI:** 10.3390/ma11010124

**Published:** 2018-01-13

**Authors:** Christian Schulze, Markus Weinmann, Christoph Schweigel, Olaf Keßler, Rainer Bader

**Affiliations:** 1 Biomechanics and Implant Technology Research Laboratory (FORBIOMIT), Department of Orthopaedics, University Medicine Rostock, Doberaner Straße 142, 18057 Rostock, Germany; rainer.bader@med.uni-rostock.de; 2 H.C. Starck Tantalum and Niobium GmbH, Im Schleeke 78-91, 38642 Goslar, Germany; markus.weinmann@hcstarck.com; 3 Chair of Material Science, Faculty of Mechanical Engineering and Marine Technology, University of Rostock, Albert- Einstein- Str. 2, 18059 Rostock, Germany; christoph.schweigel@uni-rosotck.de (C.S.); olaf.kessler@uni-rostock.de (O.K.)

**Keywords:** implant material, Ti-42Nb alloy, additive manufacturing, mechanical properties, optimization

## Abstract

The application of Ti-6Al-4V alloy or commercially pure titanium for additive manufacturing enables the fabrication of complex structural implants and patient-specific implant geometries. However, the difference in Young’s modulus of α + β-phase Ti alloys compared to the human bone promotes stress-shielding effects in the implant–bone interphase. The aim of the present study is the mechanical characterization of a new pre-alloyed β-phase Ti-42Nb alloy for application in additive manufacturing. The present investigation focuses on the mechanical properties of SLM-printed Ti-42Nb alloy in tensile and compression tests. In addition, the raw Ti-42Nb powder, the microstructure of the specimens prior to and after compression tests, as well as the fracture occurring in tensile tests are characterized by means of the SEM/EDX analysis. The Ti-42Nb raw powder exhibits a dendrite-like Ti-structure, which is melted layer-by-layer into a microstructure with a very homogeneous distribution of Nb and Ti during the SLM process. Tensile tests display Young’s modulus of 60.51 ± 3.92 GPa and an ultimate tensile strength of 683.17 ± 16.67 MPa, whereas, under a compressive load, a compressive strength of 1330.74 ± 53.45 MPa is observed. The combination of high mechanical strength and low elastic modulus makes Ti-42Nb an interesting material for orthopedic and dental implants. The spherical shape of the pre-alloyed material additionally allows for application in metal 3D printing, enabling the fabrication of patient-specific structural implants.

## 1. Introduction

The application of biomaterials, i.e., structural implants in the human body, is commonly indicated to maintain or recover lost skeletal functionality caused by tumors [1,2], malformation, fractures [3], infections, and osteolysis [4,5]. Most of the biomaterials used, about 70%–80%, are pure metals or metal alloys [6]. Titanium and its alloys, especially Ti-6Al-4V, are state-of-the-art implant materials [7] and are most frequently implanted [6]. Implants made of forged or additively manufactured Ti-6Al-4V provide very good mechanical properties and stability [8]. Due to its good biocompatibility [9], i.e., cell compatibility, which is most probably caused by a native oxidation layer on the metal surface [10,11], and its good osseointegration, Ti-6Al-4V is currently used for application in craniofacial [12], orthodontic [8,13,14,15,16], and orthopedic implants [8,15,17,18]. 

Titanium alloys usually possess higher corrosion resistance compared to commercially pure titanium [19]. Accordingly, the release of metal ions and their exposure to the human organism is reduced, resulting in fewer inflammations. Interestingly, Ti-42Nb exhibits improved cell tolerance when compared to Ti-6Al-4V, displayed by a higher cell viability of both osteoblasts and fibroblasts [20]. 

Titanium-based implants in orthopedic surgery are often directly fixed in the host bone by press fit [21,22,23,24] or screw fixation [13,24]. Thereby, the highly diverging elastic properties of the implant material, e.g., cp-Ti or Ti-6Al-4V [25] and cortical bone [26,27,28,29,30,31,32,33,34], promote stress-shielding effects along the implant–bone interphase [35,36]. As a consequence, the osseointegration of the implant, especially bone ingrowth, may be affected [13]. In the worst cases, implant loosening [36], osteolysis [4,5], and postoperative midterm migration of the implant occur [37]. 

In the past decade, additive manufacturing (AM), i.e., selective laser melting (SLM) or electron beam melting (EBM) of titanium alloys, has become an important technology for fabricating implants with complex [38] geometries that are otherwise difficult or even impossible to produce. Avoiding the constraints related to traditional production methods, AM allows for the manufacturing of implants on the basis of 3D data obtainable by standard diagnostic methods, such as computer tomography (CT) or magnetic resonance imaging (MRI). These data can then be translated via standard tessellation language (STL) into 3D print files [39,40,41].

SLM technology is applicable in various pure metals and alloys. However, the focus in recent years has been on printing stainless steel [42,43,44,45], aluminum [46], and its alloys as well as titanium [47] and titanium-based alloys, e.g., Ti-6Al-4V [48,49]. There are several promising applications in fields such as automotive, aerospace [50], medicine [51], etc., where SLM-printed parts have the potential to replace those produced conventionally. 

SLM or EBM is increasingly gaining popularity in biomedical and dental applications, especially for realizing patient-specific implant designs [52]. A special focus is on the development of biocompatible materials. This includes the ban of toxic, allergenic, or otherwise hazardous metals such as Co, Ni, Cr, Al, V, etc., and adjusting mechanical properties to the requirements of the human bone, i.e., strength and elasticity. 

Currently, the most promising materials are β-phase Ti alloys, which have been widely described in the scientific literature as having an emphasis on cast and forged materials [53,54,55,56]. They provide increased cell viability and improved mechanical compatibility with the cortical bone because of an intrinsically reduced elastic modulus when compared to Ti-6Al-4V [19,57,58,59], which crystalizes as a mixture of the α and β-phase. 

Binary Ti/Nb alloys have been well examined and understood. Above a certain niobium content, they entirely crystallize in the body-centered cubic β-phase [60]. Interestingly, the elastic modulus of Ti-Nb is stoichiometry-dependent, with two minima of ca. 68 GPa at 15 wt % of niobium (this composition crystallizes as a mixture of the α and β phase) and 62 GPa at 42 wt % of niobium [61]. Nevertheless, none of the literature discussed the AM or SLM of a spherical pre-alloyed Ti–Nb powder with a niobium content of 42 wt % that provides a minimal Young’s modulus, as described by Ozaki et al. [61] for Ti–Nb cast alloys. Some of the studies reported on in situ alloying of Ti and Nb with several content of niobium [57,59]. Prashanth et al. [25] described the mechanical properties of a porous Ti-45Nb alloy manufactured by a classic sintering process and subsequent hot pressing. The aim of our present study was the mechanical and microstructural characterization as well as the determination of the printing parameters of the Ti-42Nb alloy that was applied in the SLM manufacturing process described for the first time. Accordingly, we chose the latter composition (42 wt % of niobium) for the development of SLM powders, including powder synthesis and full powder characterization. The applicability of the newly produced spherical Ti-42Nb powders in AM was finally evaluated by SLM and the microstructure as well as the mechanical properties of manufactured 3D specimens. It could be shown that Ti-42Nb surpasses the mechanical properties of common standard implant materials, especially in terms of elasticity.

## 2. Results

### 2.1. SLM Powder Synthesis and Characterization

**Figure 1:**
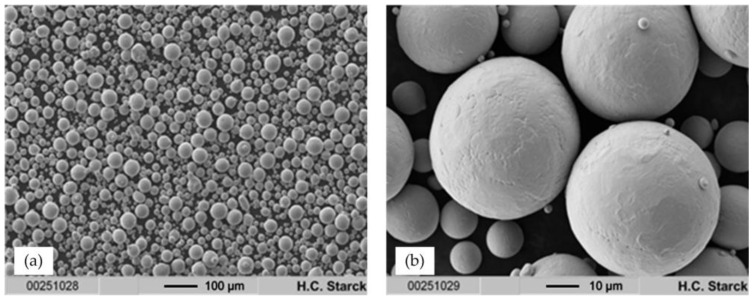
(**a**) SEM images at 100× and (**b**) 1000× magnification of AMPERTEC MAP Ti-42Nb applied for the production of compression test and tensile test specimen by means of SLM.

Ti-42Nb powders were obtained by means of electrode induction-melting gas atomization (EIGA) of pre-alloyed Ti-42Nb rods using Ar gas (4.6, Linde, Munich, Germany) for atomization. The raw powder was fractionized by sieving, and the fraction < 63 μm was used for further investigation and processing. Figure 1 indicates that the particles are highly spherical and that their surfaces are smooth without visible cracks or other faults. Accordingly, they possess ideal flow properties for application in SLM.

**Figure 2:**
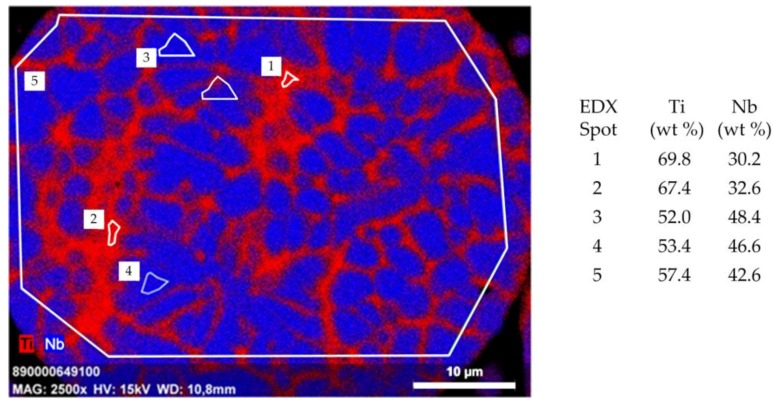
Energy-dispersive X-ray spectrometer (EDX) mapping of a 55 µm Ti-42Nb particle. The image displays a partially segregated dendrite-type microstructure with Ti-rich spots (red; 1, 2) and Nb-rich spots (blue; 3, 4). The overall composition (5) is Ti-42.6Nb and only negligibly deviating from the chemical analysis determined by ICP OES (Ti-42.5Nb; data not shown).

A representative SEM image of the polished cross section of a Ti-42Nb particle is displayed in Figure 2, indicating a dendrite-type segregation into Ti-rich and Nb-rich regions, displayed in red and blue, respectively. Pores or voids within the particle are not observed. The maximum chemical fluctuation within the as-atomized particle is in the range Ti-30Nb (spot 1) and Ti-48Nb (spot 3). The overall composition (spot 5) is Ti-42.6Nb, corresponding to the wet-chemical analysis performed using ICP-OES. According to X-ray diffraction studies, the material is entirely β-phase, whereas there is no indication for the presence of α-, α′-, or ω-phase, as reported for Ti/Nb alloys with higher Ti content [62].

### 2.2. Additive Manufacturing

Additive manufacturing of the specimens by means of SLM was performed using a Trumpf TruPrint 1000 printing system, at the Laserzentrum Hannover, Germany, applying empirically determined parameters: a laser power of 80 W, a hatch of 100 μm, and a powder feed rate (layer thickness) of 50 μm. The scan speed was 400 mm/s. Based on the empirically determined process parameters, the SLM process was carried out with a relatively low energy density of 40 J/mm^3^.

In this process, thin layers of Ti-42Nb pre-alloyed powder were homogeneously distributed on a niobium substrate plate and consolidated in x/y direction using the focused laser beam. Only those areas that were emblazed were consolidated. The substrate plate was again lowered, evenly coated with the metal powder and the layer consolidated by the laser beam. Repetition of this procedure resulted in the layer-wise formation of three-dimensional structures, i.e., compression test and tensile test specimens. A subsequent thermal treatment such as diffusion annealing or hot isostatic pressing to reduce internal stresses or porosity was not applied.

**Figure 3:**
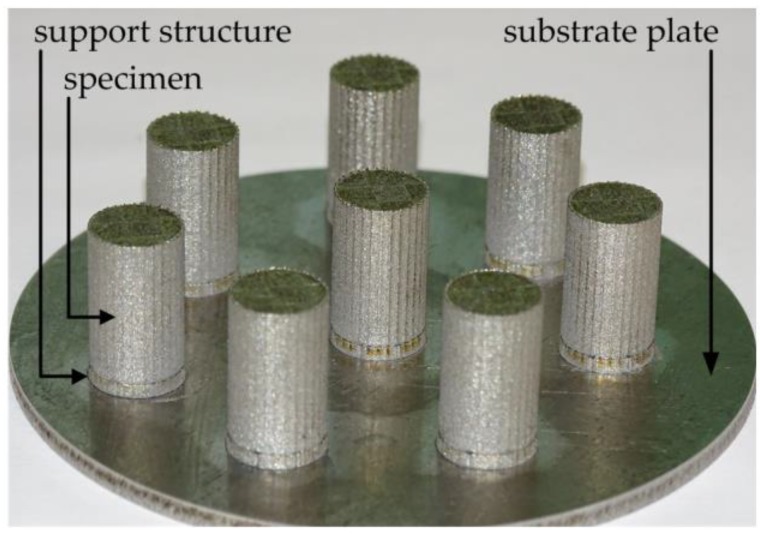
Compression test specimens in the as-built condition before separation from the substrate plate.

Figure 3 displays a set of compression test specimens printed on the above mentioned Nb substrate plate. For practical reasons, 1.5 mm thick support structures were printed initially. The printed cylinders (without support structures) had dimensions of *d* × *l* = 10.7 mm × 16 mm. After removal from the base plate, they were machined to *d* × *l* = 6.9 mm × 10.35 mm (*d*:*l* = 1.5) for further testing. Tensile test specimens with as-printed dimensions of *d* × *l* = 15 mm × 70 mm were obtained in accordance to the removal process of the compression test specimens. The final dimensions of the tensile test specimens in the “as-machined” condition are shown in chapter Materials and Methods.

**Figure 4:**
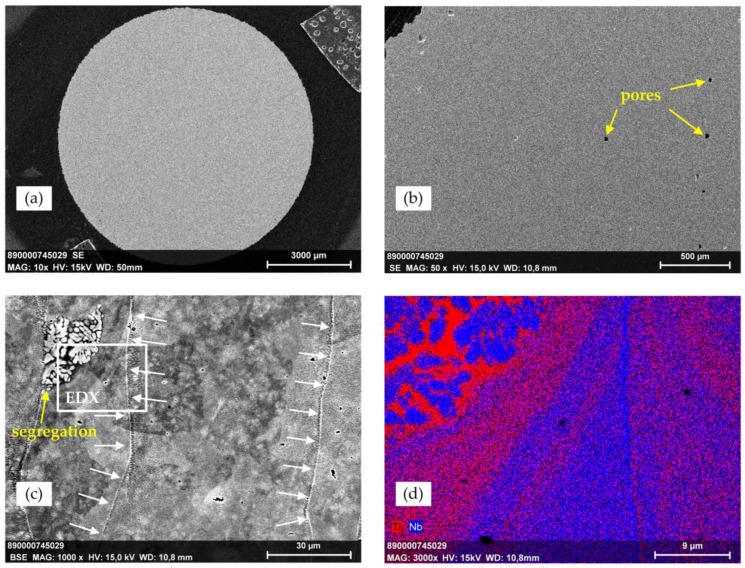
SEM/EDX images of a polished surface of a Ti-42Nb tensile test specimen at (**a**) 10×; (**b**) 50× and (**c**) 1000× magnification in BSE mode; (**d**) The EDX mapping at 3000× magnification arises from the clip in the BSE image.

Figure 4 demonstrates SEM images of a polished surface of an as-printed tensile test specimen, a BSE image as well as an EDX mapping. The overview image (a) points to the fact that the as-printed specimen is homogeneously densified. Larger pores, voids, or segregation of large-scale inhomogeneities are not visible. On the basis of image (b), the relative density of the specimen can optically be estimated to be >99.5%. The BSE image (c) likewise displays a dense matrix that is rarely interspersed by tiny pores having diameter in the micron range. As a main feature, grain boundaries are visible (white arrows). The segregation in the upper left corner of the BSE image can undoubtedly be attributed to a Ti-42Nb particle or the residue of such a particle, which has not been melted by the laser beam during the SLM process. This is strongly supported by the EDX mapping (d), which displays the dendrite-type structure of the spherical SLM powder particles shown in Figure 2. However, such inhomogeneities are only extremely rarely observed throughout the specimens. 

Most importantly, the entire microstructure of the applied material dramatically changed during the SLM processing. It is evident that the initial dendritic microstructure of the precursor powder completely vanishes during the SLM process, most probably to the extreme cooling rates between 10^4^ and 10^6^ K/s [63]. Accordingly, the constituting elements Ti and Nb are dispersed homogeneously on a sub-micron scale.

**Figure 5:**
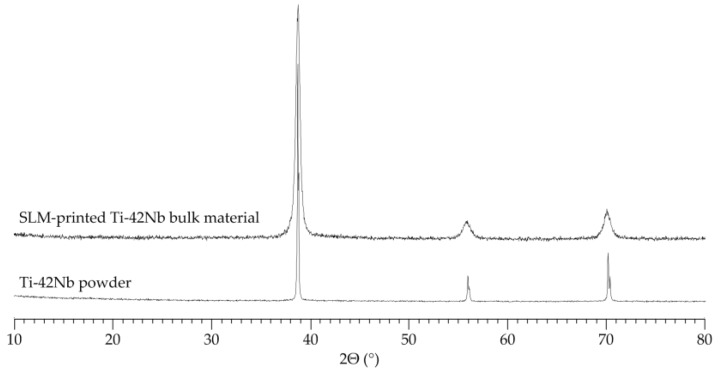
Results of the X-ray diffraction analysis performed for the Ti-42Nb precursor powder and the SLM-printed Ti-42Nb bulk material.

While Ti-rich Ti/Nb alloys may form complex microstructures composed of mixtures of α, α′, α′′, ω and/or β phase [64,65,66,67], X-ray diffraction (XRD) studies of SLM-printed Ti-42Nb (Figure 5) clearly indicate that the material entirely crystallizes in a bcc β-phase. This is in accordance with results obtained by XRD investigations using cast Ti-42Nb. Interestingly, a comparison of the XRD patterns of pre-alloyed precursor powder and SLM-printed Ti-42Nb display notably different line widths. The reflections of the printed material are markedly broadened compared to those of the powder, suggesting a significantly finer microstructure, which forms as a consequence of the extremely fast cooling during SLM processing.

### 2.3. Mechanical Characterization

**Figure 6:**
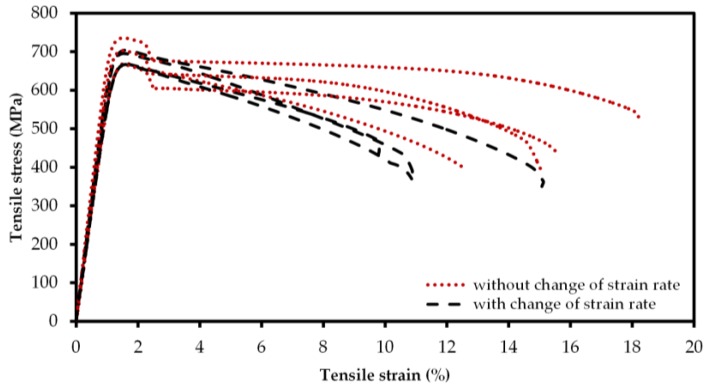
Stress-strain curves of the tensile test specimens tested with higher strain rate (black dashed) and with lower strain rate (red dotted) in the plastic region. All the determined stresses and strains are engineering stresses and strains. Specimens tested with higher strain rate in the plastic region showed higher decrease in stress with increasing tensile strain, whereas at the lower strain rate, the fractures occurred at higher tensile strains.

All tensile test specimens (*n* = 8) showed nearly equal slope in the elastic regime. For the SLM-printed Ti-42Nb tensile specimens, an average Young’s modulus E = 60.51 ± 3.92 GPa was computed. During the tensile tests, no distinct yield strength could be observed. Thus, the proof strength at 0.2% plastic extension σ_0.2_ was ascertained at 674.08 ± 24.77 MPa from the stress-strain curves. Due to the lack of distinct yield strength, the point of the 0.2% plastic extension (elongation) was chosen to switch the strain rate from ė_1_ (0.00025 s^−1^) to ė_2_ (0.0067 s^−1^) for determination of the ultimate yield strength (UTS) in the plastic region of the material. In four of the eight tensile specimens, the universal testing machine detected a rapid decrease in stress directly after reaching σ_0.2_ (Figure 6). Thus, the strain rate was not changed from ė_1_ to ė_2_ for these specimens (specimen numbers 1; 3; 6; 7), and only four specimens (specimen numbers 2; 4; 5; 8) could be considered for the determination of the ultimate yield strength UTS. An ultimate yield strength of 683.17 ± 16.67 MPa (*n* = 4) without distinct densification was observed in the tensile tests. For specimens tested with the higher strain rate (ė_2_) in the plastic region fracture strength of 384.49 ± 31.36, MPa was determined at an elongation at fracture of 11.65% ± 2.03%, whereas specimens tested with lower strain rate (ė1) showed fracture strength of 435.29 ± 58.22 MPa for elongation at fracture of 15.37% ± 2.00%. Thereby, the latter showed higher variance of stress in the plastic region. 

**Figure 7:**
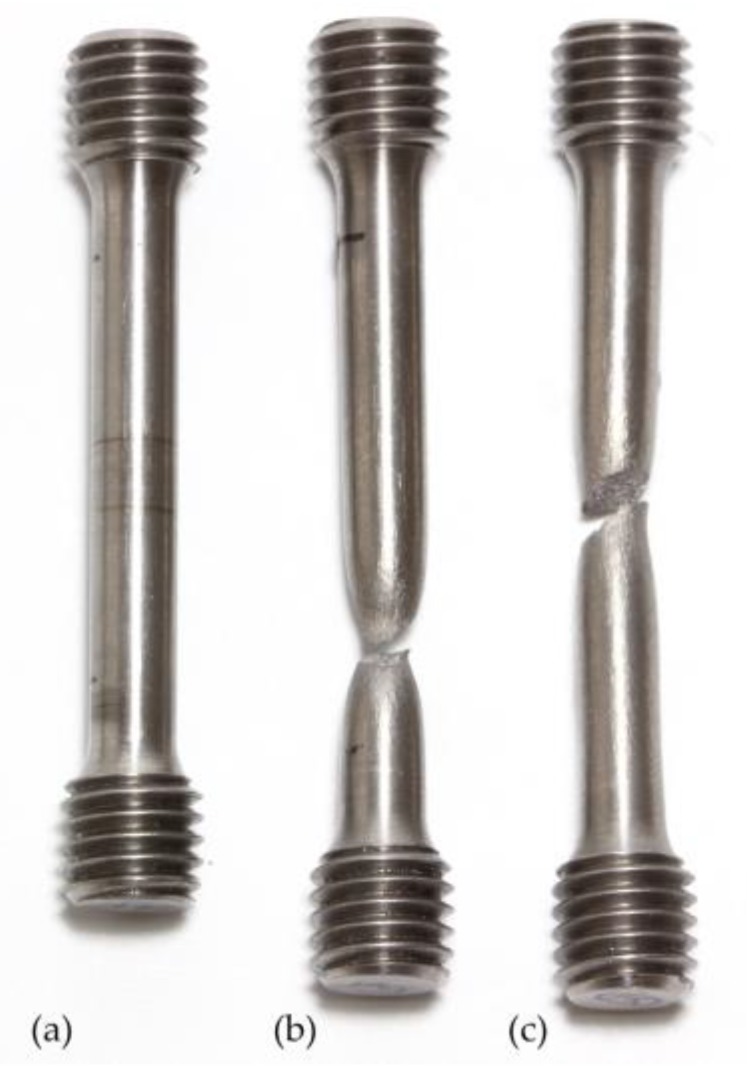
Exemplary depiction and visual comparison of the tensile test specimens according to ISO 6892-1 before (**a**) and after (**b**,**c**) tensile tests. Seven of the eight fractured specimens markedly displayed the typical necking (**b**), whereas one of the specimens (**c**) showed shear fracture.

All specimens tested displayed the typical fracture appearance of ductile materials (cup-and-cone fractures) (c.f. Figure 7b). The determination of the necking at the fracture spot revealed nearly equal necking behavior for specimens, independent of whether they were tested with strain rate ė_1_ and ė_2_ (44.17% ± 4.52%) or with constant strain rate ė1 (42.59% ± 7.02%). One of the eight specimens, i.e., specimen 3, which was tested with lower strain rate (ė_1_), revealed a shear fracture resulting in lower necking (31.40%) than specimens with cup-and-cone shaped fractures (Figure 7c).

In Table 1, the mechanical properties determined in tensile tests of the SLM-printed Ti-42Nb are summarized and compared to Ti alloys, commonly employed as implant material. The comparison of the mechanical properties of the Ti-42Nb alloy under tensile load and the mechanical properties of human cortical bone is shown in Table 2.

**Figure 8:**
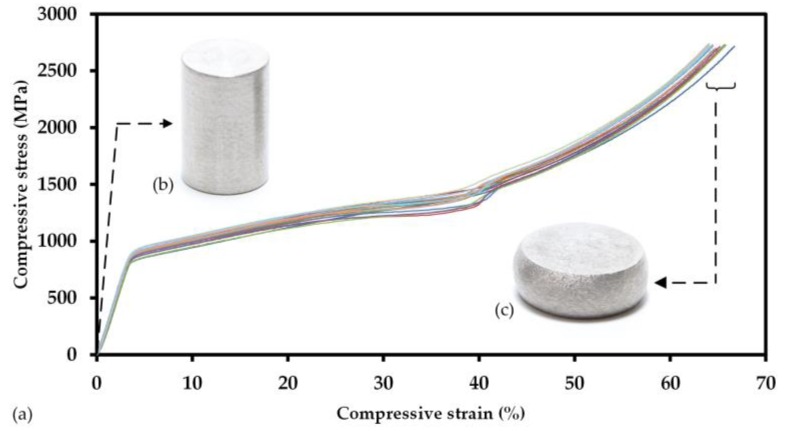
(**a**) Stress-strain curves of 17 analyzed compression test specimens. The more or less identical slopes of the stress-strain curves of all samples suggest a consistent SLM-process, independent of the position of the specimens on the building platform. The inserts correspond to compression test specimens before (**b**) and after (**c**) application of a compressive load of 101.9 kN.

All Ti-42Nb specimens for compression tests (*n* = 17, specimen numbers 9; 10; 12–26) showed similar plastic material behavior in the uniaxial compression test. One specimen was identified as aberration (specimen 11) and was excluded from the calculation of the mean values and standard deviations of the investigated parameters. All specimens were compressed until the maximum load of 101.9 kN was applied, resulting in about 65% compression of the specimens; thereby, no fractures could be observed (Figure 8). The mean diameter enlargement of the specimen at maximum load was 4.37 ± 0.34 mm. 

Under compressive loading, Ti-42Nb revealed the lack of a distinct yield limit for which reason the offset yield strength at 0.2% compression σ_c 0.2_ = 831.58 ± 30.11 MPa and offset yield strength at 2% compression σ_c 2_ = 921.36 ± 31.07 MPa was determined. The compression yield strength σ_c 35_ = 1330.74 ± 53.45 MPa was determined at the predefined compression of 35%. The compression modulus was not determined due to the fact that the compression tests were not carried out with an additional external extensometer. Table 2 shows the determined mechanical properties of Ti-42Nb and the mechanical properties of cortical bone found in the literature. 

**Figure 9:**
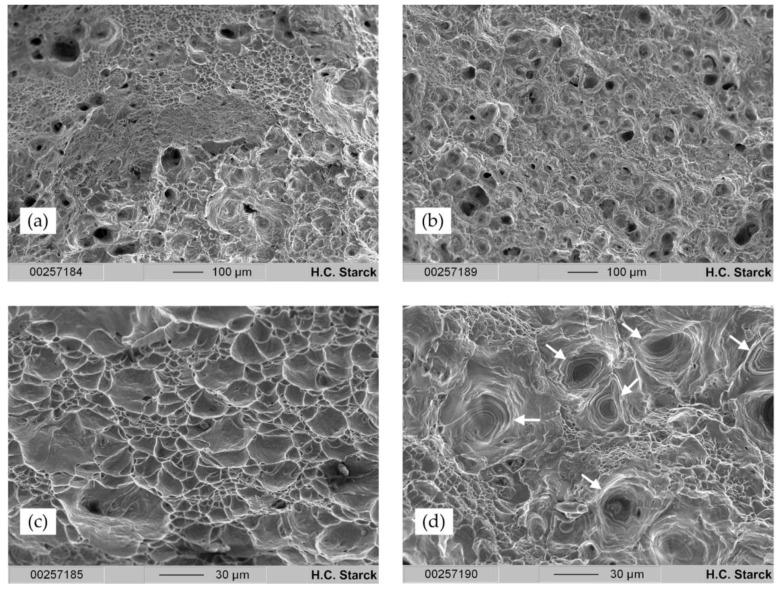
Scanning electron microscope images of the fracture spots after tensile testing of the Ti-42Nb tensile specimens at a 100× (**a**,**b**) and 500× (**c**,**d**) magnification for specimen 2 (**a**,**c**) and specimen 3 (**b**,**d**). The fracture surface of tensile specimen 2 (**c**) displays a comb structure typical for ductile material failure. Voids with circular lines marked by the white arrows in specimen 3 (**d**) indicate the presence of defective structures in the printed specimen.

The SEM analysis of the fracture surfaces of specimen 2 (tested with ė_1_ to ė_2_) and specimen 3 (Figure 9) revealed two different fracture mechanisms. As discussed, specimen 2 showed a cup-and-cone fracture, whereas as an exception, specimen 3 failed due to shear fracture. The shear fracture may arise from internal imperfections; however, there could currently no internal imperfections be determined in the microstructure of the tensile test specimens, excluding the segregation shown in Figure 4d. The fracture surface of specimen 2 displayed a comb-like structure with combs in different sizes. These features clearly indicate an entirely ductile material behavior of SLM-printed Ti-42Nb specimens. In contrast, the SEM investigations into the fracture surface of specimen 3 revealed circular voids with fracture lines highlighted by the white arrows in Figure 9d. These spots are about 30 µm in diameter. However, as observed for those samples with typical cup-and-cone fracture, a comb-type structure with very small combs can be observed between these circular spots.

**Figure 10:**
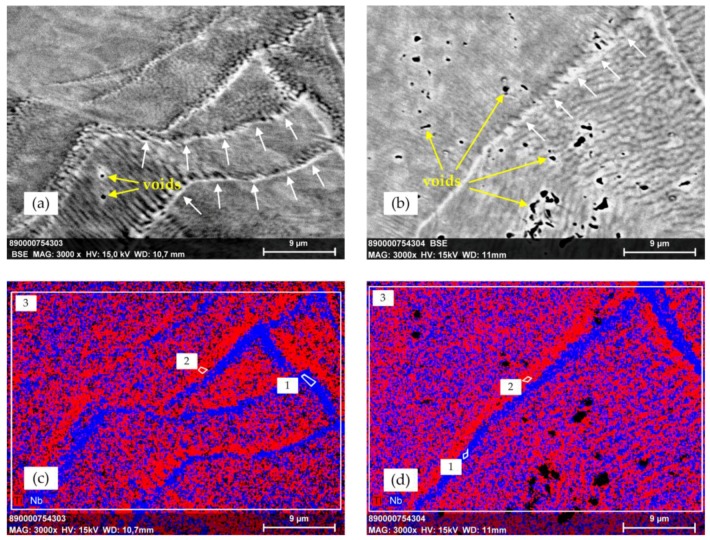
Scanning electron microscope images (**a**,**b**; BSE mode) and energy dispersive X-ray spectrometer (EDX) mapping (**c**,**d**) of polished cross-sections of Ti-42Nb compression specimens 10 (**a**,**c**) and 3 (**b**,**d**) after compression tests at 3000× magnification. The images (**a**,**b**) display structures that may be identified, for e.g., grain boundary formation and sliding bands, respectively, indicated by white arrows. The encountered structure sizes are in the range of few microns. For specimen 10 (**a**), only a few very small voids with diameter in the sub-micron range are observed, whereas a recurrent pattern of voids (up to 2 µm) is found for specimen 11 (**b**). The images (**c**,**d**) display a partially de-mixed microstructure with Ti- (red) and Nb-rich spots (blue). A spatial separation of Ti and Nb along the encountered structures is ascertained in both samples.

To get insights into the microstructure of Ti-42Nb specimens under compression, SEM/EDX investigations were performed on the polished surfaces of compression test specimens. SEM (BSE mode)/EDX investigations of the tested compression specimens displayed a formation of structures that are not completely understood yet (Figure 10). Compression specimens showed mostly dense morphology. However, in specimen 10 very small pores with diameter in the sub-micron range could be observed, whereas in compression specimen 11, greater pores up to 2 µm were found with recurrent patterns. Along the encountered structures that may be for e.g., grain boundaries or sliding bands, slight demixing is observed with a local enrichment of Ti (indicated in red color) and Nb (blue color). The chemical composition of Ti and Nb measured with EDX from measuring spots 1 to 3 (Figure 10) is given in Table 3.

Apart from the encountered structures with slight enrichment of Ti and Nb, a microstructure with very homogeneous distribution of Nb and Ti could be observed, whereas there was no indication of the presence of dendrite-type structures, as observed in the powder. EDX scans over the entire particle area (spot 3) exactly mirrored the results of the element analysis.

## 3. Discussion 

The difference in elastic modulus between implant material and host bone leading to bone loss due to stress shielding has been extensively discussed in the literature [72,73,74,75]. Ti alloys are widely used implant materials with sufficient biocompatibility. However, α- and α + β-type Ti alloys, which are commonly applied in AM, possess relatively high elastic moduli [74] when compared to entirely β-type Ti alloys. Accordingly, they may cause stress-shielding and associated implant loosening [55,76,77]. Accordingly, we describe the synthesis and SLM technology of spherical Ti-42Nb with the goal to produce 3D bulk material with an elastic modulus much closer to that of the human bone than standard Ti alloys. Test specimens manufactured by SLM (as-built) were used for tensile and compression tests. The material characterization was supported by SEM/EDX analysis of the newly produced alloy powder. SEM revealed a significant change in the microstructure of the powder after the laser melting process. Furthermore, the characterization of mechanical properties showed decreased elastic modulus and good ductility when compared to the commonly employed implant materials [8,34]. 

The main limitation of the present study is the inherent simplification of the loads applied to the materials in mechanical testing in comparison to combined loads in vivo appearing due to complex force situation [78]. Thereby, bending and torsional loads are predominantly [78,79] applied in vivo to orthopedic implants, e.g., the femoral component of total hip endoprostheses. However, torsion and bending test were not conducted to characterize the mechanical properties of Ti-42Nb under such loads. Furthermore, the material behavior under cyclic loading and the fatigue resistance have not been investigated so far. In the tensile test set up applied within this study in four of eight specimens the UTS was determined at a strain rate of ė_1_ = 0.00025 s^−1^. In general, the UTS should be determined with a strain rate of ė_2_ = 0.0067 s^−1^ [80] due to omitted change from strain rate ė_1_ to ė_2_. This certainly enables the assessment of the UTS under different load rate conditions. In contrast, the compression test was conducted without an external extensometer. Thus, the validity of the measured force-displacement behavior in the elastic region of the material is narrowed; due to this, the compressive modulus was not computed. All specimens were tested in the as-machined condition. Additional heat treatment was not performed due to the fact that the mechanical characterization of the Ti-42Nb alloy newly applied in additive manufacturing should be conducted in the initial condition (“as machined”). Nevertheless, it is expected that a heat treatment of the printed specimens, such as hot isostatic pressing (HIP) and/or diffusion annealing, will lead to reduced internal stresses. However, either of these treatments will release a coarser-grained microstructure when compared to the as-printed state and most probably result in increased elongation at fracture and a decrease in UTS. The analysis of the microstructure by means of SEM and EDX could only be conducted at two of the tensile test specimens and only three of the compression test specimens. The segregation that indicates the dendrite-like structure of the Ti-42Nb precursor powder in Figure 4d was the only observed particle residue in all analyzed specimens independent from the direction of the polished cross section.

The dendrite-like microstructure in the precursor Ti-42Nb particles, which was observed in EDX investigations, was not maintained during the SLM processing. Against this, the printed parts displayed a microstructure with a very homogeneous distribution of niobium and titanium. This pointed to the fact that the melt generated by the laser rapidly cooled down under the applied fabrication conditions; literature values reported cooling rates during SLM processes of about 10^6^ K/s [63]. Accordingly, fast temperature dissipation during the laser melting process resulted in a mostly homogenous distribution of Ti and Nb on a sub-micrometer scale apart from the structures that may be identified, e.g., grain boundaries and sliding bands, respectively. An enrichment of Ti and Nb appeared solely in these structures. 

The density of 99.5%, which was estimated visually from the cross section of the Ti-42Nb specimens made by SLM, is in agreement with the observations of Yasa et al. [81] for SLM-manufactured Ti-6Al-4V specimens. The solid material structure was indicated by the presence of only a very small amount of round-shaped weak features within the x–y plane of the as-printed tensile specimens. No large pores were observed, as reported by Vandebrouke et al. [52], for low energy input by the laser beam. Nonetheless, laser molten material contained fewer pores and showed finer microstructures when compared to cast materials [52]. 

All raw cylinders had been printed with the same height, and only the position on the substrate plate (initial x-y-plane) varied. In the polished cross section (x-y-plane) of the printed bulk material cylinders, a homogenous distribution of niobium and titanium on a sub-micron scale was determined using EDX-analysis. In a preliminary investigation, electron backscatter diffraction (EBSD) of SLM-printed Ti-42Nb was used to determine the predominant grain orientation parallel and perpendicular to the building direction (pictures are not shown). The results revealed (i) the existence of very fine microstructures with (ii) the orientation of elongated grains in z, i.e., in the building direction.

All specimens were machined along the building direction out of the raw cylinders to match the specimen dimension claimed by the DIN 50106 standard (tensile specimens) [82] and DIN 50125 standard (compression specimens) [83]. Both the standards demand a polished surface of the effective measure length l_0_; due to this recommendation, we decided to machine the specimens out of the printed bulk material. Parameters such as surface roughness, building direction as well as global position of the printed part in the powder bed and the influence of these parameters on the mechanical properties should be investigated in further studies. 

Wysocki et al. [84] reported that the UTS of SLM-printed CP Ti varied with the position of the printed part on the building platform, whereas the porosity changed with the height of the printed parts. It was furthermore reported that the building direction of the parts had an influence on the elongation at fracture under tensile loading [84]. The graduated porosity along the building direction and the different elongation in both cross sections had an effect on the necking of the sample [84].

It was observed that tensile test specimen 3 fractured at 45° in relation to the load direction and circular voids with fracture lines at the fracture surface. The reason for the formation of these features is not understood at the moment. Most probably they arise from pores introduced during the SLM process. Several reasons may be attributed to their formation, as reported by Zhang et al. [85], for example, increased solubility of gas in the melt, trapped gas bubbles in the melt pool, or insufficient powder bed density (“voids” in the powder bed). The other seven among eight samples showed typical cup-and-cone fractures, and the typical comb-type structure could be observed. In contrast, no fractures were observed during the compression tests of the β-type Ti-42Nb alloy. Nevertheless, Chlebus et al. [86] described the occurrence of shear fracture in compression tests of α + β-type Ti-6Al-7Nb alloy.

Mechanical testing of the SLM-manufactured β-type Ti-42Nb alloy confirmed the findings of Ozaki et al. [61] that the Young’s modulus is about 62 GPa at a concentration of 42 wt % of niobium, which was determined for cast material. Furthermore the prediction of a distinct lower modulus for β-stabilized Ti alloys [6] compared to α-type Ti and α + β-type Ti alloys [6] could be approved in the present study. However, even though the UTS of SLM-printed Ti-42Nb is markedly higher than that of CP Ti, it is distinctly reduced about 71% when compared to Ti-6Al-4V. Both Young’s modulus and the strength of SLM-printed Ti-42Nb compare well to those of conventionally manufactured, i.e., forged Ti-42Nb [61]. SLM-printed Ti-6Al-4V, which has been developed as a high-strength alloy for application in aerospace has a markedly higher ultimate strength (1075 MPa in the building direction) and a about 80% higher elastic modulus (115–119 MPa in the building direction) [38]. Eventually, an additional heat treatment may optimize the UTS [87] of the Ti-42Nb alloy, but this will be part of follow-up studies. However, this issue is discussed controversially. For example, Leuders et al. showed that heat treatment considerably above the β-transus of Ti-6Al-4V increased the elongation at fracture with a coincident decrease of UTS [49]. 

The Young’s modulus of SLM-printed Ti-42Nb determined under tensile load is 2.5 to 4.4 times higher than that of the cortical bone of the femur [26,27,70] and the tibia [70]. A high difference in the elastic modulus between the implant and the bone is associated with insufficient load transfer [53] leading to eventual bone resorption and loosening of the implant [53]. Nonetheless, the observed modulus of the β-type Ti-42Nb is only about half the modulus provided by Ti-6Al-4V. Such an increase in elasticity is expected to prevent stress-shielding, which in the worst case is associated with bone mass loss and implant loosening. Furthermore, adjusting the intrinsic properties of a material is not the only approach to reduce the stiffness of implant structures. Structural behavior and stiffness of the implants can be influenced by the grade of porosity and the geometry of the applied structural elements [88,89,90]. Disadvantage of porous structures is the reduced fatigue resistance when compared to solid materials. Apart from this due to the high stiffness of commonly used metallic implant materials the size of the unit cells and the resulting porosity relative to the diameter of the struts have to be enlarged to match the mechanical and structural properties of human bone. This may affect the ingrowth of the bone cells into porous structures if the unit cell size exceeds more than 600 µm [91,92]. With a SLM-printed Ti-42Nb material that provides about half the stiffness of the commonly used bone implant materials based on Ti alloys the unit cell size can be better adjusted to the mechanical properties of human bone.

## 4. Materials and Methods 

### 4.1. Powder Synthesis 

Ti-42Nb spherical powder (Trade name AMPERTEC MAP Ti-42Nb) is a newly developed commercial product distributed by H.C. Starck. It is obtained by electrode induction-melting gas atomization (EIGA) of Ti-42Nb rods using purified Ar (4.6, Linde). The SLM fraction is refined by ultrasonic sieving through 63 µm stainless steel meshes and subsequent sifting to remove particles with diameter < 5 µm. AMPERTEC MAP Ti-42Nb is almost 100% spherical; satellites or irregular non-spherical particles are rarely observed (**Error! Reference source not found.**). The particle surfaces are smooth without visible cracks or other faults. This is confirmed by PSD (Master Sizer 2000 (Malvern, Worcestershire, UK); not shown), which displays a monomodal distribution with D(10) = 28.20 μm, D(50) = 44.80 μm and D(90) = 69.61 μm.

### 4.2. Specimen Manufacturing

**Figure 11:**
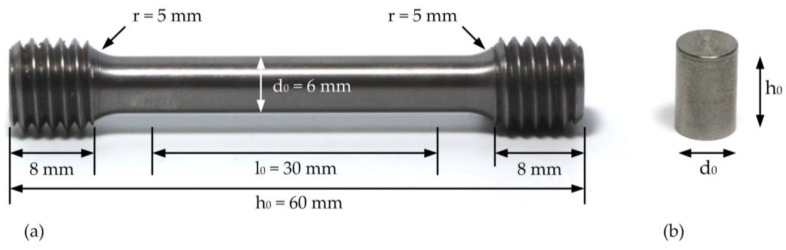
(**a**) Tensile test specimen after CNC machining the as-built specimens to the dimension claimed by the DIN 50106 standard; (**b**) compression test specimen after separation from the substrate plate and CNC-machining to the dimensions claimed by the DIN 50125 standard. The surface quality of the tensile and compression specimens is a result of the machining process and was not additionally polished.

3D-manufacturing by means of SLM was performed using a Trumpf TruPrint 1000 printing system (TRUMPF GmbH + Co. KG, Ditzingen, Germany; 200 W fibre laser; wavelength ~1070 nm) at Laserzentrum Hannover (LZH), Germany. Parameters were determined empirically under variation of laser power (40–120 W) and scan speed (80–500 mm/s). The hatch width was 100 µm and the powder feed rate was 50 µm/layer. Based on microstructural investigations of polished cross-sections of 4 × 4 × 4 mm^3^ test cubes, a laser power of 80 W and a scan speed of 400 mm/s were chosen for printing specimens applied in compression and tensile strength tests. The specimens were layer wise printed with a square scanning pattern. Post heat treatments such as diffusion annealing to reduce internal stresses or hot isostatic pressing to remove closed pores were not applied. The sizes of the rods printed for compression and tensile strength testing are provided in Table 4. The raw cylinders were subsequently CNC-machined to match the specimen dimensions according to DIN 50125 specimen shape B [83] for tensile test and according to DIN 50106 [82] for the compression test (Table 4; Figure 11).

### 4.3. Mechanical Test Setup

**Figure 12:**
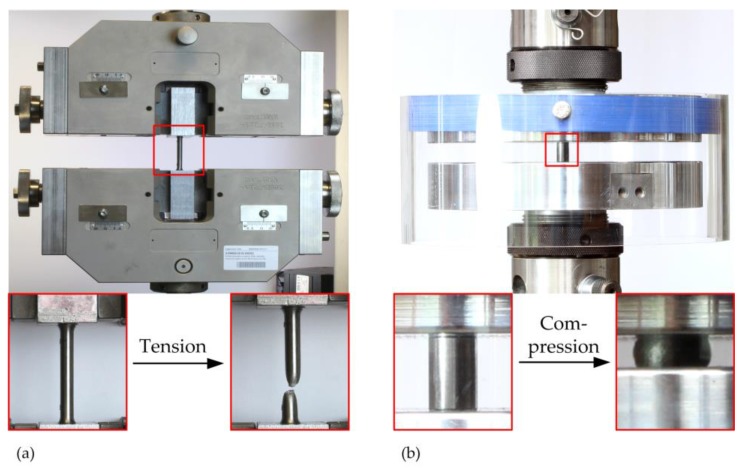
(**a**) Test setup for the characterization of the mechanical properties under tensile (**a**) and compressive load (**b**). (**a**) In the tensile tests, the specimen elongation was recorded by a video extensometer; (**b**) Highly polished compression plates applied uniaxial compression until the maximum of 101.9 kN compressive load was reached.

The mechanical properties of the Ti-42Nb alloy were determined in tensile and compression tests according to EN ISO6892-1 [80] and DIN 50106 standard [83]. Tensile tests were performed on a universal testing machine Z050THW (Zwick GmbH & Co. KG, Ulm, Germany) and the compression tests were conducted on a servo-hydraulic testing machine Instron 8502 (Instron Deutschland GmbH, Pfungstadt, Germany) at room temperature (Figure 12).

Uniaxial tensile test of the Ti-42Nb alloy was carried out until the fracture of the specimens (*n* = 8, specimen 1–8) occurred; meanwhile, the specimen elongation was recorded by the optical extensometer videoXtens (Zwick GmbH & Co. KG, Ulm, Germany). The parameters, Young’s Modulus E and proof strength at 0.2% plastic extension σ_0.2_, were determined at a strain rate of ė_1_ = 0.00025 s^−1^ based on the elongation measured with the extensometer. For determination of the ultimate tensile strength (UTS), the strain rate was switched from ė_1_ to ė_2_ = 0.0067 s^−1^ after the plastic extension at 0.2% was reached. Furthermore, the uniaxial compression tests for the Ti-42Nb specimens (*n* = 18, specimen 9–26) were carried out at a cross speed of 30 MPa/s until the predefined maximum load of 101.9 kN was reached. During the compression test, the reaction force was recorded, and the shortening of the compression specimens were determined by a displacement of the compression stamp. The compression test was conducted without an external extensometer. The compressive load was applied by highly polished compression plates on the specimens. Subsequently the compressive strength at 35% strain σ_c 35_ and the offset yield strength at 0.2% strain σ_c 0.2_ and 2% strain σ_c 2_ were computed based on the compression-stress compression-strain relation. Due to the lack of cracks or fractures of the specimens during the compression test, the compressive strength was analyzed at 35% compressive strain. All the determined stresses and strains are engineering stresses and strains.

### 4.4. Chemical and Microstructural Characterization

For investigation of the structural characteristics of the raw Ti-42Nb powder material and the SLM manufactured test specimens, the scanning electron microscopy (SEM; Jeol JSM 6490 LV, Akishima, Tokyo, Japan) in combination with energy dispersive X-ray (EDX; Bruker Quantax EDS with SDD Detector (Bruker, Berlin, Germany), resolution 130 eV; recording time 200 s, ca. 1.5 Kcps, excitation voltage 28 KV; quantification standardless via ZAF routine) was performed. The chemical composition (Ti- and Nb-content in wt %) of the powder was evaluated on a polished 55 µm AMPERTEC MAP Ti-42Nb particle at five measuring spots. The characterization of the chemical composition of the compression specimens was conducted for specimens 10 and 11 after the compressive load was applied. Therefore, compression specimen 10 was polished perpendicular to the loading direction and compression specimen 11 was polished to the cross section in the direction of the applied load. For both specimens, the local chemical composition was measured at spots 1 and 2. The averaged chemical composition was determined at spot 3. Furthermore, the fracture surfaces of the tensile specimen 2 and 3 were exemplarily analyzed by SEM investigation. The fracture of the SLM-manufactured Ti-42Nb alloy was characterized and assessed with regard to the pores and fracture behavior. In addition, X-ray diffraction studies were performed using a PANalytical X-ray diffractometer (Almelo, Overijssel, Netherlands) (X’Pert MPD-PRO, Cu Kα radiation, 2Θ = 10–80°)

## 5. Conclusions

Ti-42Nb has been developed as an alternative implant material. The focus was on lowering the Young’s modulus when compared to standard implant materials to improve the elastic compatibility with human bone. Another objective was to develop Ti-42Nb with spherical morphology to allow for a fabrication of patient-specific implants using additive manufacturing, i.e., powder bed-based 3D printing processes. Ti-42Nb spherical particles were obtained by electrode induction-melting gas atomization (EIGA) of pre-alloyed Ti-42Nb rods and successfully 3D printed using selective laser melting (SLM). As-printed bulk materials possessed densities > 99.5% and microstructures with extremely homogeneous element distribution of Ti and Nb. Tensile strength test revealed significantly improved elasticity of SLM-printed Ti-42Nb (ca. 60 GPa in tension) when compared to CoCr (235 GPa), CP Ti (ca. 105 GPa) or Ti-6Al-4V (ca. 110 GPa). The proof strength at 0.2% plastic extension of Ti-42Nb was determined at ca. 675 MPa, which is lower than that of Ti-6Al-4V (790–870 MPa) but significantly higher than that of CP Ti. In conjunction with its excellent biocompatibility published recently, Ti-42Nb seems to be a very promising material for application in orthopedic and dental implants in the near future.

## Figures and Tables

**Table 1:** Mechanical properties of selected Ti-based implant materials determined in tensile tests.

Material	Manufacturing	Condition	E (GPa)	σ_0.2_ (MPa)	UTS/UCS (MPa)	ε_b_ (%)
CP-Ti [8]	-	Grade 1–4	102.7–104.1	170–485	240–550	15–24
Ti-6Al-4V [8]	annealed	-	110–114	825–869	895–930	6–10
Ti-6Al-4V ELI [8]	mill annealed	-	101–110	795–875	860–965	10–15
Ti-6Al-7Nb [8]	-	-	114	880–950	900–1050	8.1–15
Ti-6Al-4V [34]	EBM	as built	118 ± 5	830 ± 5	915 ± 10	13.2 ± 0.6
		hipped	117 ± 4	795 ± 10	870 ± 10	13.7 ± 1.0
		wrought/annealed	104 ± 2	790 ± 20	870 ± 10	18.1 ± 0.8
Ti-6Al-4V [17,68,69]	SLM	-	112	990–1330	1095–1407	2–5
Ti-45Nb [25] (compression test)	green compacted + sintered	-	18.5 ± 2.5	-	282 ± 26	-
	hot-pressed	-	64.4 ± 9.4	-	667 ± 56	-
	Hot-pressed + sintered	-	60.3 ± 8.9	-	686 ± 64	-
Ti-26Nb * [57] (* at %; compression test)	Ingot	-	69.9 ± 0.2	-	-	-
	SLM (in situ alloyed)	-	77 ± 1.4	-	-	-
Ti-27.5Nb * [58] (* at %)	CLAD^®^-Process	as built	70 ± 3	-	-	-
		Solution treated	70 ± 4	-	-	-
Ti-42Nb (present study)	SLM	CNC-machined	60.51 ± 3.92	674.08 ± 24.77	683.17 ± 16.67	11.65 ± 2.03

**Table 2:** Mechanical properties of human bones of different anatomic regions and the comparison with Ti-42Nb specimens made SLM-printed.

Material	Test Method	Anatomic Region	E (GPa)	σ_0.2_ (MPa)	UTS/UCS (σ_c 35_) (MPa)	ε_f_ (%)
Cortical Bone	Tensile	Femur [26,27,70]	13.6–16.8	-	68–141	1.07–2.83
		Tibia [70]	16.2–23.83	-	84–157	1.56–3.09
	Compression	Tibia osteons [71]	22.5–25.8	-	-	-
		Femur [70]	17.6	-	194	-
		Tibia [70]	28	-	195	-
Ti-42Nb (present study)	Tensile	-	60.51 ± 3.92	674.08 ± 24.77	683.17 ± 16.67	11.65 ± 2.03
	Compression	-	-	831.58 ± 30.11	1330.74 ±53.45	-

**Table 3:** Chemical composition of compression test specimens 10 and 11 at different locations determined by SEM/EDX.

EDX Measuring Spot	Specimen 10		Specimen 11	
	Ti (wt %)	Nb (wt %)	Ti (wt %)	Nb (wt %)
1	55.7	44.3	56.3	43.7
2	59.1	39.9	58.1	41.9
3	57.7	42.3	57.5	42.5

**Table 4:** Specimen dimension according to DIN 50106 (compression specimens) and DIN 50125 (tensile specimens).

Specimen	As Printed		As Machined		As Measured	
	d (mm)	h (mm)	d_0_ (mm)	h_0_ (mm)	d_0_ (mm)	h_0_ (mm)
Tensile (Specimen 1–8)	15.0	70.0	6.0	60.00	5.98 ± 0.01	60.06 ± 0.05
Compression (Specimen 9–26)	10.7	16.0	6.9	10.35	6.89 ± 0.02	10.33 ± 0.03
